# Corrigendum: Application of a multispecies probiotic reduces gastro-intestinal discomfort and induces microbial changes after colonoscopy

**DOI:** 10.3389/fonc.2024.1395635

**Published:** 2024-03-27

**Authors:** Joachim Labenz, Daniela-Patricia Borkenstein, Franz Josef Heil, Ahmed Madisch, Ulrich Tappe, Harald Schmidt, Birgit Terjung, Ingeborg Klymiuk, Angela Horvath, Manfred Gross, Vanessa Stadlbauer

**Affiliations:** ^1^ Department of Internal Medicine, Diakonie Klinikum Jung-Stilling, Siegen, Germany; ^2^ Praxis Dr. Heil und Dr. Müller, Andernach, Germany; ^3^ Department of Internal Medicine I, Hospital Clinic Siloah, Hannover, Germany; ^4^ Centrum Gastroenterologie Bethanien, Agaplesion Krankenhaus Bethanien, Frankfurt, Germany; ^5^ Gastropraxis an der St. Barbara Klinik, Hamm, Germany; ^6^ Praxis für Innere Medizin und Gastroenterologie Dr. H. Schmidt, Berlin, Germany; ^7^ GFO Kliniken Bonn, St. Josef-Hospital, Bonn, Germany; ^8^ Division of Cell Biology, Histology and Embryology, Gottfried Schatz Research Center, Medical University of Graz, Graz, Austria; ^9^ Division for Gastroenterology and Hepatology, Department of Internal Medicine, Medical University of Graz, Graz, Austria; ^10^ Area 3 Microbiome Modulation for Precision Medicine, Center for Biomarker Research in Medicine (CBmed), Graz, Austria; ^11^ Department of Internal Medicine, Internistisches Klinikum München Süd, Munich, Germany

**Keywords:** adverse events, colonization, colonoscopy, colorectal cancer, prevention

A correction has been made to **Material and methods, *Participants, multispecies probiotic and placebo formulation*, paragraph 1.**

This sentence previously stated:

“The probiotic group received the multispecies probiotic formulation containing 3g of Bifidobacterium bifidum W23, Bifidobacterium lactis W51, Enterococcus faecium W54, Lactobacillus acidophilus W37, Lactobacillus rhamnosus WGG, Lactococcus lactis W19 at a concentration of 9x10^9^ cfu/g in a matrix of rice starch, maltodextrin, hydrolyzed rice protein, potassium chloride, manganese sulfate and magnesium sulfate (OMNi BiOTiC^®^ Colonize) twice daily over 30 days.”

The corrected sentence appears below:

“The probiotic group received the multispecies probiotic formulation containing 3g of Bifidobacterium bifidum W23, Bifidobacterium lactis W51, Enterococcus faecium W54, Lactobacillus acidophilus W37, Lactobacillus rhamnosus WGG, Lactococcus lactis W19 at a concentration of 3x10^9^ cfu/g in a matrix of rice starch, maltodextrin, hydrolyzed rice protein, potassium chloride, manganese sulfate and magnesium sulfate (OMNi BiOTiC^®^ Colonize) twice daily over 30 days.”

A correction has been made to **Discussion**, paragraph 10. The sentence previously stated:

“In conclusion we demonstrated in a randomized, placebo controlled, double-blind study conducted in a homogenous population undergoing screening colonoscopy that the application of a multispecies probiotic at a concentration of 2x10^9^ cfu/g with 6 g per day starting right after colonoscopy, ameliorates side effects of colonoscopy induced constipation significantly.”

The corrected sentence appears below:

“In conclusion we demonstrated in a randomized, placebo controlled, double-blind study conducted in a homogenous population undergoing screening colonoscopy that the application of a multispecies probiotic at a concentration of 3x10^9^ cfu/g with 6 g per day starting right after colonoscopy, ameliorates side effects of colonoscopy induced constipation significantly.”

The authors apologize for these errors and state that this does not change the scientific conclusions of the article in any way. The original article has been updated.

