# Supplementary figures and images for: Application of a multispecies probiotic reduces gastro-intestinal discomfort and induces microbial changes after colonoscopy
Source: Front Oncol. 2023 Jan 9;12:1078315. doi: 10.3389/fonc.2022.1078315 (PMC9870247; doi:10.3389/fonc.2022.1078315)

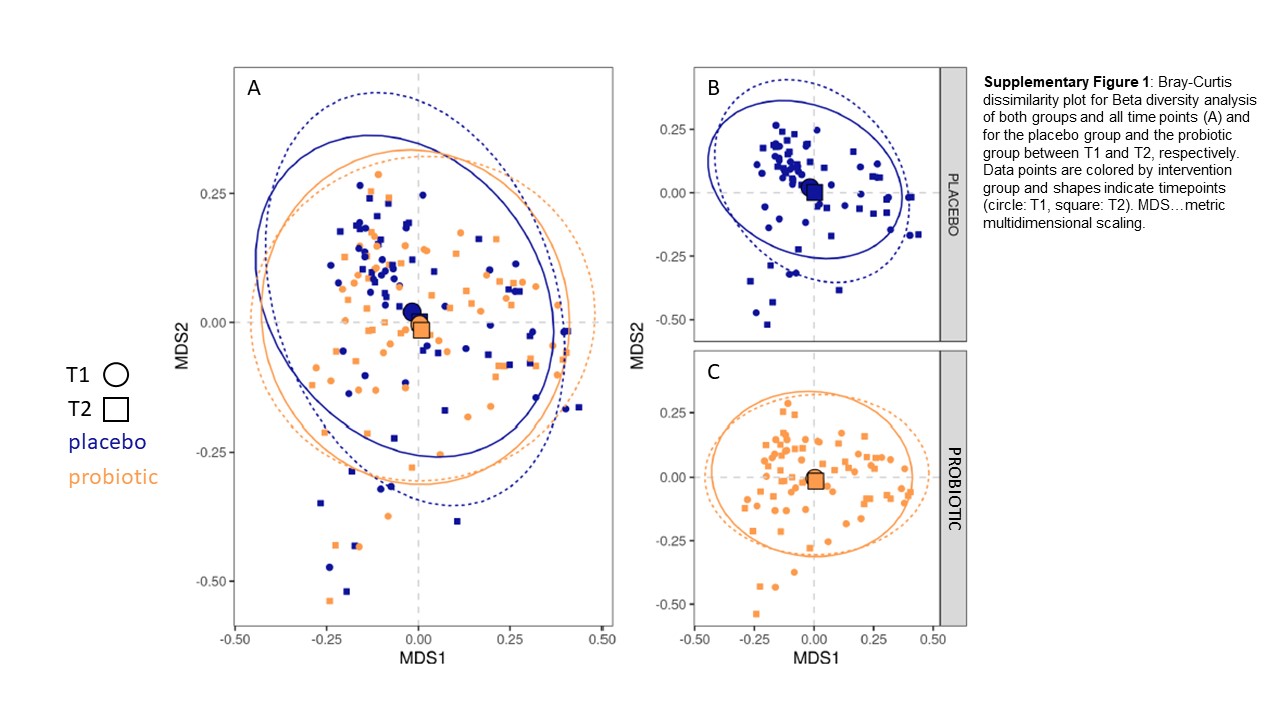

Supplement: Supplementary file 1 [file Image_1.jpeg]

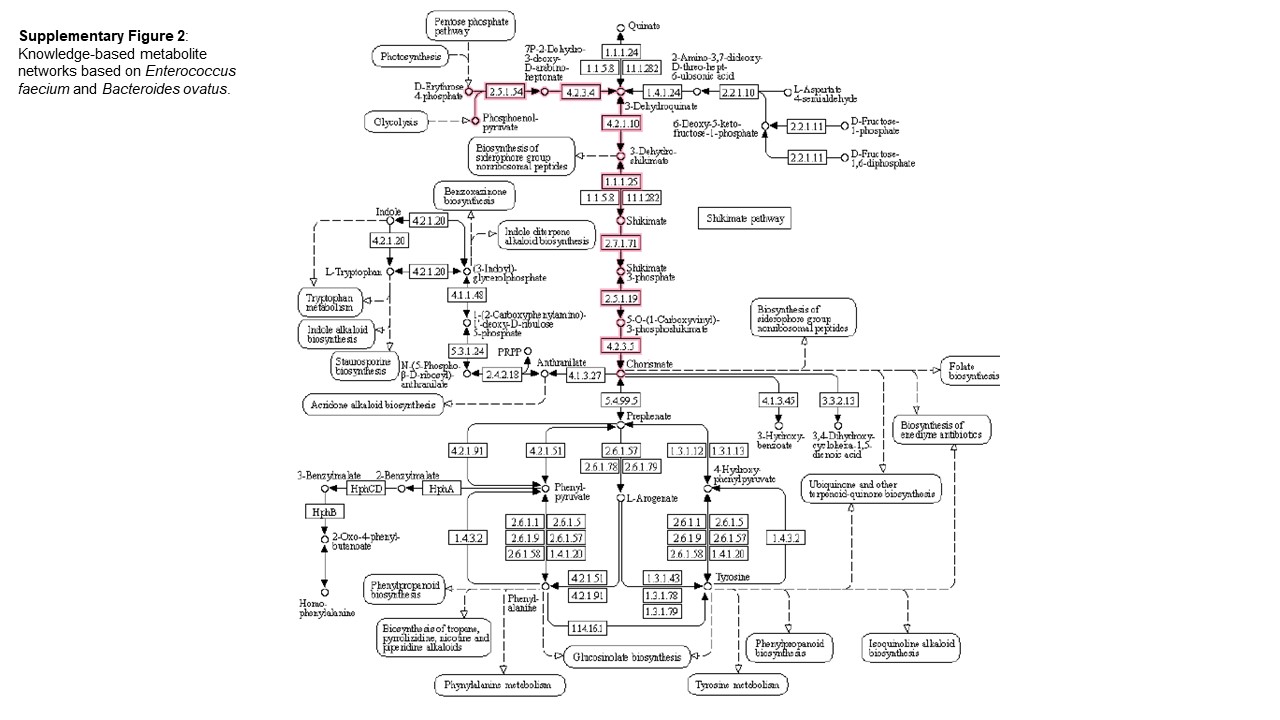

Supplement: Supplementary file 2 [file Image_2.jpeg]
